# Harnessing the Effect of Adoptively Transferred Tumor-Reactive T Cells on Endogenous (Host-Derived) Antitumor Immunity

**DOI:** 10.1155/2010/139304

**Published:** 2010-11-07

**Authors:** Yolanda Nesbeth, Jose R. Conejo-Garcia

**Affiliations:** ^1^Department of Microbiology and Immunology, Dartmouth Medical School, Lebanon, NH 03756, USA; ^2^The Immunology Program, The Wistar Institute, Philadelphia, PA 19104, USA

## Abstract

Adoptive T cell transfer therapy, the ex vivo activation, expansion, and subsequent administration of tumor-reactive T cells, is already the most effective therapy against certain types of cancer. However, recent evidence in animal models and clinical trials suggests that host conditioning interventions tailored for some of the most aggressive and frequent epithelial cancers will be needed to maximize the benefit of this approach. Similarly, the subsets, stage of differentiation, and *ex vivo* expansion procedure of tumor-reactive T cells to be adoptively transferred influence their *in vivo* effectiveness and may need to be adapted for different types of cancer and host conditioning interventions. The effects of adoptively transferred tumor-reactive T cells on the mechanisms of endogenous (host-derived) antitumor immunity, and how to maximize their combined effects, are further discussed.

## 1. Introduction

It has been more than 50 years now since Thomas and Burnet first proposed the hypothesis that the immune system could identify and eradicate transformed or malignant cells, confirming earlier observations by Paul Ehrlich that an “overwhelming frequency” of carcinomas could be repressed by the immune system. This intrinsic ability of the immune system to provide control against malignancies has since been refined and termed immunosurveillance [1–4]. Despite the presence of immunosurveillance properties within the immune system, immunocompetent patients still develop cancers, yet these tumors are often less immunogenic than those that develop in immunosuppressed hosts. These and other observations led to the demonstration that tumors are imprinted by their immune environment, and this imprinting facilitates their transformation into populations that can more effectively resist the pressure exerted by the immune system to eradicate them [5–7]. This process, in which the immune system acts both positively to inhibit the progression of tumors and negatively to mold the establishment of tumors that can evade its recognition, or worse to promote the advancement of tumor development, is referred to as immunoediting [3, 8]. Thus, the immune system can prevent or promote tumor progression.

## 2. Myeloid Leukocytes Accumulate at Tumor Locations and Induce Immunosuppression

 Professional antigen presenting cells (APCs) with adequate stimulatory capacity are necessary within the tumor microenvironment (TME) to induce sufficient effector cells or cytokines to maintain their tumor-fighting capacity. However, tumor-bearing hosts do not appropriately present tumor antigens. Instead, they mobilize immature myeloid cells that include precursors of macrophages, dendritic cells (DCs), and neutrophils. These cells, generically termed Myeloid-Derived Suppressor Cells [9] (MDSCs), massively accumulate at splenic and solid tumor locations, where they contribute to tumor progression by providing growth factors, as well as paracrine support for the formation of blood vessels [10–15]. Most importantly, MDSCs abrogate antitumor immune responses through multiple mechanisms that include, at least, the production of L-Arginase, NO and reactive oxygen species [10, 16–22], and the tyrosine nitration of the T cell receptor [23]. Because of the heterogeneous nature of the precursors recruited to tumor locations as immature MDSCs, more differentiated but still immunosuppressive macrophages or dendritic cells are also frequently found in the tumor microenvironment. In tumors, the precise categorization of myeloid cells is therefore complicated by a high degree of phenotypic overlap and also depends on specific microenvironments. In ovarian cancer, for instance, we have repeatedly demonstrated that the most abundant leukocyte subset in the SOLID tumor microenvironment in humans, and in both tumor masses and ascites in mice, expresses low but detectable levels of phenotypic markers of *bona fide* DCs, including CD11c, DEC205, CD86, and MHC-II (10, 13–15, 22, 24, 25). Irrespective of their overlapping phenotypic characterization, we have repeatedly demonstrated that when these tumor leukocytes receive specific activating signals, they can functionally process full-length OVA *in vitro *[14, 24] and *in vivo *[22, 25], as well as effectively present processed SIINFEKL to T cells [10, 15, 22, 25].

Yet, while DCs are also abundant in the microenvironment of many other tumors, functional mature DCs capable of stimulating an antitumor response are not found in high frequencies in human breast cancer, prostate cancer, ovarian cancer, or renal cell carcinoma [26–30]. Cancer cells produce various factors such as VEGF [31–36] and IL-6 [31, 37] that suppress DC differentiation and maturation [38, 39]. At the same time, cytokines that promote DC differentiation, such as granulocyte–macrophage colony-stimulating factor (GM-CSF) and IL-4 and Th1 polarizing cytokines like IFN-*γ* and IL-12, are seldom found in large quantities in many human cancers, and ovarian cancer in particular [40, 41]. Thus, this skewed cytokine profile promoted by the tumor impairs the effective priming of an immunostimulatory DC phenotype and promotes the transition of DC precursor cells recruited to the tumor microenvironment into a suppressive population. Importantly, in several cancer systems, DCs in both the tumor microenvironment and peripheral blood can revert to an immunostimulatory phenotype *in vitro* and can prime tumor-specific T cell responses [40, 42]. Nonetheless, the modulation of APCs does not appear to be strong enough to overcome the tolerogenic environment of many tumors. In fact, in ovarian cancer, patients receiving multiple rounds of fully matured myeloid DCs were not able to regain T cell function after their *in vivo* association with suppressive tumor-associated plasmacytoid DCs [40].

## 3. T Cells Exert Spontaneous Immune Pressure against Cancer Progression

 In contrast, despite the heterogeneous nature of the CD3^+^ T cell compartment, the presence of T cells in the various malignancies generally correlates with improved clinical outcomes to the point that CD3^+^ T cells are considered the only immune population capable of exerting antitumor effects against established tumors [43, 44]. 

The evidence of immune cell infiltrates and their ability to mount antitumor responses in various tumor systems have led investigators to target tumors through modulation of the immune response. Immune-based therapies are delivered either through active immunotherapy, in which vaccines such as peptides, tumor antigens, nucleic acids, engineered tumor cells, or tumor-pulsed DCs are used to activate host antitumor immune cells to react against the tumor, or passive immunity wherein antibodies or antitumor lymphocytes are transferred into tumor-bearing hosts to directly induce tumor cell destruction [45]. Passive immunotherapy has revealed high success rates in certain implications, however, as most protocols direct responses against a single antigen/epitope, and tumors often modulate their expression of particular antigens, there is often a high degree of inefficacy. Active immunotherapy in both mouse and human tumor systems have resulted in potent antitumor responses and regression, and is beneficial in the fact that rather than restricting responses to a single epitope /antigen, polyclonal responses can readily be induced. 

While both forms of immunotherapy have demonstrated positive results, they each have drawbacks. The ideal system would entail passive therapeutics that can immediately start eliminating the tumor while inducing an active endogenous response to continue the tumor eradication. Under ideal circumstances, transferred T cells could migrate to the tumor site and directly lyse tumor cells while releasing endogenous immune cells from the tumor-induced immunosuppression. However, the tumor environment is usually so immunosuppressive that it is difficult to appropriately release these brake mechanisms on antitumor responses.

## 4. Adoptive Cell Transfer Therapy Induces the Rejection of Advanced Tumors

 Adoptive cell transfer therapy (ACT), the ex vivo activation, expansion, and subsequent administration of tumor-reactive T cells, is a vastly successful therapy against certain cancers. In fact, ACT is currently the most effective therapy against metastatic melanoma, with objective regressions reported in 50% of patients [46–49]. Adoptive T cell therapies have focused on the use of CD8^+^ T cells, as they have relatively long clonal expansion times, can specifically target tumors, and are easily subjected to genetic manipulations. Lymphodepletion has been used to enhance the persistence of transferred T cells *in vivo.* By eliminating suppressive populations, removing cytokine sinks-endogenous cells that compete with the transferred cells for cytokines that promote their activation and function, and through augmenting the function and availability of APCs, lymphodepletion is thought to enhance the antitumor response. In fact, in melanoma, ACT was only effective after prior lymphodepletion of patients, and this combination produced distinct and reproducible responses in roughly 50% of melanoma patients being treated with ACT. 

ACT has also displayed remarkable success in human clinical trials against Epstein-Barr virus- (EBV-) related disorders, immunoblastic lymphoma, and also Non-Hodgkin's disease [45, 50–55]. Yet, although these findings are optimistic for the future of adoptive immunotherapy, these systems are markedly different in that they are virally induced tumor systems, and the T cells are directed against foreign, rather than self, antigens. In most malignancies, being nonviral, T cell antigenic targets are often self-antigens. This further complicates the ability to produce large numbers of tumor-reactive T cells since, not only do they usually occur in only low frequencies [56], but also most T cells that robustly respond to self antigens have either been eliminated during thymic development or rendered nonfunctional by local tolerizing mechanisms [57–59]. In fact, T cell adoptive therapies have not resulted in impressive clinical benefits yet against the most lethal-epithelial-tumors [60–62]. Therefore, the expansion protocols for transferred T cells need to maximize both the quality and quantity of tumor-reactive T cells produced. As such, much work has gone into identifying strategies to optimize the ex vivo expansion of tumor-reactive T cells for ACT.

## 5. Ex Vivo Generation of Tumor-Reactive T Cells for Adoptive Transfer

 The main sources of modulation of the conditions for T cell expansion include the antigen source, the cytokine environment, and the source and effector stage of the T cells before expansion [57]. Various adoptive cell transfer regimens entail the nonspecific, polyclonal expansion of T cells through mitogenic stimulation as with phytohaemagglutinin, or antibodies to CD3/CD28. These nonspecific manipulations have achieved significant response rates against hepatocellular carcinoma, myeloma, non-Hodgkin's lymphoma, and Hodgkin's disease [45, 63–66]. This would indicate that not only do the direct tumorlytic effects of CD8^+^ T cells contribute to the beneficial responses of ACT, but also that secreted factors may also play a role.

The expansion of T cells against tumors of nonviral origin pose different challenges, including the low frequency of CTLs against self antigens [45]. In humans, the low frequency of precursor populations of tumor-reactive T cells has been circumvented by prior vaccination of patients with helper peptide-based vaccines. Vaccination of breast and ovarian cancer patients with HER-2/neu peptide-based vaccines supplemented with GM-CCSF adjuvant treatment over a six-month period increased the precursor frequency of HER-2/neu-specific T cells that were capable of secreting IFN-*γ* in response to tumour and directly lyse HER-2/neu expressing tumors [67]. Similarly, vaccination of breast cancer patients with MUC-1 helper peptide vaccines produced CTLs reactive against MUC-1 expressing tumors [68]. Recent mouse models of melanoma have shown that these time-consuming and often cumbersome vaccination strategies may be bypassed by appropriate *in vitro* programming of the transferred T cells. These protocols have an additional benefit over helper peptide vaccination in that they can facilitate the expansion of polyclonal lymphocyte cultures.

An innovative approach used to circumvent the low frequency of tumor-reactive T cells in cancer patients has been the genetic manipulation of autologous T cells to express either T cell receptors (TCR) targeted to tumor-associated antigens (TAA), or with chimeric receptors encompassing a B cell receptor to a particular antigen complexed with the TCR signaling domain (so-called T-bodies) [48, 69]. In a phase I clinical trial, administration of allogeneic T cells that recognize the HLA-A2-restricted peptide MART-1 induced a partial response in one patient, while remaining patients did not yield any overall response [70]. In another study, autologous peripheral blood lymphocytes retrovirally transduced with the MART-1 TCR induced complete regression in two patients with metastatic melanoma, but had no effect on the remaining 13 patients in the cohort [48]. While this technique provides a method for bypassing the customary low numbers of tumor-specific T cells capable of being harvested from cancer patients, it poses the problem of limiting the potential antitumor response to a single epitope which, if downregulated by the tumor, would render the procedure useless. 

Alternatively, T cells may be modified with genes to inhibit the induction of apoptosis or senescence. The antiapoptotic genes BCL-2 and BCL-x_L_ have been introduced into T cells resulting in the extended survival of such cells even under conditions that would usually promote apoptosis [71, 72]. Recently, it was demonstrated that both mouse and human tumor-reactive T cells could be effectively transduced with siRNA to downmodulate their expression of PD-1. As anticipated, this prevented inhibitory signaling through the PD-1/PD-L1 inhibitory pair and instead generated T cells with enhanced proliferation and immune function as determined through IL-2 and IFN-*γ* secretion [73]. This technique provides access to a new realm of ACT, wherein T cells can be engineered to specifically avoid the debilitating TME without the need to induce systemic methods for disrupting immunosuppression. 

Other manipulations include the introduction of autocrine growth signals into T cells before transfer to enhance their *in vivo* proliferation. This was first attempted through the overexpression of IL-2 on T cells, which had no effect on the tumorlytic capacity of these transferred T cells. In contrast, unlike IL-2, IL-15 does not promote the expansion of Tregs and when overexpressed in human T cells prolonged the expression of antiapoptotic genes thus the persistence of tumor specific cells and enhanced their antitumor responses [74, 75].

## 6. Relative Efficacy of Individual T Cell Differentiation Subsets

 The therapeutic effects of ACT have been commonly attributed to the *in vivo* expansion and antitumor activity of transferred lymphocytes. Correspondingly, major efforts have been focused on promoting long-term persistence of adoptively transferred T cells [76]. Various *in vitro* manipulations have been evaluated to enhance the *in vivo* persistence of transferred T cells. Thus, the T cells have been cultured in the presence of, or coadministered with, the cytokines IL-2, IL-7, IL-12, IL-15, and IL-21 [77, 78]. While each has been found to have its own specific benefits and demonstrated to enhance the therapeutic effects of adoptive immunotherapy in mouse models of melanoma, their effects have been related to the promotion of a specific effector phase of the transferred T cell. This has led to several studies evaluating the efficacy of particular T cell differentiation subsets in adoptive T cell therapy against cancer. 

Although effector memory T cells (T_EM_) are superior to central memory T cells (T_CM_) at inducing *in vitro* cytotoxicity of transformed cell cultures, they have poor replicative capacity *in vivo,* and T_CM_ exert superior therapeutic benefits to T_EM_ cells [76, 79–82]. Central memory T cells, being the least differentiated of the antigen experienced population of T cells, and being thought to have the capacity for self-renewal, and for retaining the option to differentiate into a vast repertoire of T cell populations, was for some time considered the ideal starting population of cells for expansion protocols [83]. These T_CM_ cells can undergo robust expansion in response to secondary exposure to antigen and secrete copious levels of IL-2, in stark contrast to T_EM_ cells. It was later found that compared with more differentiated effector lymphocytes, or memory T cells, early effectors have a higher capacity for *in vivo* expansion, which is associated with enhanced therapeutic effects against melanoma [84]. Thus, fewer early effector T cells specific to the gp100 antigen were necessary to induce regression of melanoma in mice. More recently still, we demonstrated for the first time that naïve or briefly activated T cells can induce potent antitumor responses in adoptive T cell transfer experiments, which was subsequently confirmed in an independent study [24, 85, 86]. These effects coincided with the in vivo differentiation of these precursor cells into cytotoxic cells and the induction of endogenous immune responses, which were found to be necessary for the therapeutic effects.

## 7. Tumorlytic Activity of CD4^+^ versus CD8^+^ T Cells

 Most investigations on the antitumor effects of T cells have centered around CD8^+^ T cells due to their high expression in various malignancies, ease of isolation and *in vitro* manipulation, and their keen ability to directly lyse tumor cells [87]. Several reports had demonstrated the efficacy of CD8^+^ T cells in inducing potent antitumor responses, although it is widely accepted that their ability to clear tumors requires further manipulation of the T cell directly, for example, through genetic elimination of inhibitory surface receptors [88], addition of specific TCRs or of the hosts through irradiation [89], or other immune interventions. Still, the contribution of CD4^+^ T cells to adoptive immunotherapy, particularly against epithelial cancers, remains controversial. However, it is widely accepted that a great deal of the failures that arise from the use of CD8^+^ T cells stem from the absence of CD4^+^ T cell help necessary for maintaining their *in vivo* functionality [90–93]. Despite the immense amount of data supporting the positive contribution of certain subsets of CD4^+^ T cells in enhancing the efficacy of function and persistence of CD8^+^ T cells, manipulations utilizing CD4^+^ T cells have been very limited. CD4^+^ T cells present as a particularly difficult population of cells to work with as they do not proliferate as effectively *in vitro* as do CD8^+^ T cells [94], and very little progress has been made in the identification of class II restricted peptides [87]. Furthermore, most tumors do not express MHC-II and are therefore not directly recognized by CD4^+^ T cells. 

Due to the great degree of homogeneity within the CD4^+^ T cell compartment and thus the wide spectrum of opposing effects potentially inducible by these cells, as well as the deficit in knowledge of MHC-II (CD4) restricted epitopes [87], the role of CD4^+^ T cells in antitumor immunity remains an investigative area that has been largely neglected. Furthermore, the majority of studies into this population have focused on the adverse effects of regulatory CD4^+^ T cells, thus creating a negative reputation for these cells in tumor immunology. Still, there have been several reports demonstrating the beneficial role of CD4^+^ T cells in antitumor immunity, providing rationale for undertaking further investigations in this area. 

Evidence from various studies show that in the absence of CD8^+^ T cells, CD4^+^ T cells were still capable of eliminating both haematologic and solid tumors [95–97]. Using transgenic T cells specific to different H-Y antigens, Perez-Diez and colleagues were able to demonstrate in 6 different tumor models that CD4^+^ T cells were more effective than CD8^+^ T cells (or a mixed population of both CD4^+^ and CD8^+^ T cells) at rejecting tumors even in the absence of MHC-II expression on the tumor cells [98]. There exists the possibility that the differences in antigen epitopes and TCR avidities may be responsible for these observed effects. Importantly, however, the authors found that antigen presentation by host cells was required at the effector phase for this tumor rejection by primed CD4^+^ T cells and speculate that this may be through the activation of local macrophages and other cells but never validated this. 

Recently, two articles confirmed the positive contribution of CD4^+^ T cells in adoptive immunotherapy against melanoma, both describing a direct tumorlytic effect of these transferred T cells [85, 99]. In both cases, small numbers of naïve CD4^+^ T cells specific to the Trp1 melanoma antigen were transferred into irradiated recipient mice bearing established B16 melanoma. Interestingly, these cells expanded robustly and importantly differentiated into cytotoxic CD4^+^ T cells that directly eliminated B16 melanomas [85, 99]. These tumors do not express MHC-II, but it was further shown that the secretion of IFN-*γ* by the transferred CD4 led to the upregulation of MHC-II on these tumors making them direct targets of the transferred T cells. In one context, further immune intervention by antibody-mediated blockade of the coinhibitory receptor CTL-associated antigen 4 (CTLA-4) on T cells augmented the antitumor activity through enhancing the expansion of the transferred T cells, increasing IFN-*γ* levels, thus cytotoxicity, and reducing the number of Tregs present.

## 8. Alternative Mechanisms of Enhanced Antitumor Immunity Mediated by CD4^+^ T Cells

 While the studies referred to above underscore the direct tumorlytic potential of CD4^+^ T cells, it is generally accepted that most human tumors do not express MHC-II and are therefore insensitive to CD4^+^ T cell-mediated cytolysis. However, other than directly lysing tumors, CD4^+^ T cells have been demonstrated to contribute to antitumor responses through the provision of cytokine support, the maintenance and survival of CD8^+^ T cells and through the expression of CD40L [100–104]. Indeed, we demonstrated that adoptively transferred CD4^+^ T cells, through CD40L-CD40 interactions, license tumor-associated DCs to prime endogenous antitumor CD8^+^ T cells [24, 86]. Thus, DCs that in the tumor microenvironment contributed to the promotion of immunosuppressive conditions, when given the appropriate stimuli, including CD40 signaling through CD40L- expression on transferred T cells, were capable of priming antitumor responses. This concurs with their ability to uptake tumor antigens while retaining an immature phenotype such that the mere provision of this additional stimulus was capable of reversing their phenotype. This induction of endogenous responses had greater ramifications, as we demonstrated that these host immune responses remained active for prolonged periods and protected naïve mice from challenge with the same tumor [24, 86].

In addition to directly promoting CD8^+^ T cell functionality, CD4^+^ T cells have been shown to secrete various cytokines that activate host antigen presenting cells, and their coadministration with CD8^+^ T cells revealed enhanced therapeutic benefits coupled with the induction of a robust central memory response [105–107]. Thus, Hunder et al. provided evidence with a single case of effective adoptive T cell therapy utilizing NY-ESO-1-specific CD4^+^ T cells cultured with IL-7 and IL-2 for the treatment of a patient with metastatic melanoma who had not received prior lymphodepletion or vaccination therapy [49]. 

Moreover, the infiltration of immune populations in ovarian cancer is modulated by chemokines, which therefore influence the clinical outcome. Elucidation of factors that contribute to the infiltration of immune cells into the ovarian cancer microenvironment (but not breast cancer) [108–110], revealed that tumors with significant T cell infiltrates had elevated levels of various chemokines, including CCL5, the production of which was found to be restricted to the lymphocyte population rather than the tumor cells [111, 112]. Our studies demonstrate that CD4^+^ T cells expanded against tumor antigen secrete high levels of CCL5, thus promoting the recruitment of CCR5 expressing T cells and DCs to the tumor site [24, 86]. The chemokine receptor CCR5 is expressed on memory/effector like T cells and is associated with Th1 type responses. Our findings have been mirrored by a report from Dobrzanski et al. that demonstrates that the adoptive transfer of MUC1 specific CD4^+^ T cells increase endogenous T cell activity and the survival of patients with residual recurrent epithelial ovarian cancer, and that these effects corresponded with increased expression of CCR5 and associated ligands on tumor responsive T cells [113]. 

Collectively, these data indicate that CD4^+^ T cells contribute positively to the induction of antitumor responses achieved through adoptive T cell transfer regimens in ovarian cancer, and likely in other tumors. We found that CD4^+^ T cells could independently delay tumor progression but a mixed population of CD4^+^ and CD8^+^ T cells induced greater antitumor efficacy against our aggressive model of ovarian cancer. Thus, we now appreciate the fact that it is the quality rather than quantity of adoptively transferred T cells that is more relevant for achieving positive clinical outcomes, and that the appropriate host conditioning strategies must be employed to retain their functionality and maximize their therapeutic efficacy.

It should be noted, however, that preliminary results from ongoing trials in patients with metastatic melanoma suggest that the inclusion of antitumor CD4^+^ T cells in the adoptively transferred T cell population results in poorer clinical responses, which are associated with the expansion of the regulatory T cell compartment [114]. It is therefore likely that the antitumor effectiveness of CD4^+^ T cells could depend on the type of cancer or the host conditioning strategy applied to support the adoptively transferred lymphocytes. For instance, high doses of IL-2 are administered to patients receiving antitumor T cells, but not always to tumor-bearing mice in these published reports. The preferential effect of IL-2 on regulatory T cells contained among the CD4^+^ T cells could at least partially explain the discrepancies between mouse systems and these clinical results, and help to design improved approaches.

## 9. Immunosuppressive Tumor Microenvironmental Networks Abrogate the Activity of Adoptively Transferred Tumor-Reactive T Cells against Aggressive Epithelial Tumors

 Adoptive T cell therapy, while highly successful for many nonepithelial cancers, has not yet been effective in the most frequent and aggressive epithelial cancers, likely due to the peculiarities of their respective microenvironments. In ovarian cancer, for instance, adoptively transferred autologous T cells directed at the *α*-folate receptor disappeared rapidly (often within a month) in association with increasing levels of an undetermined inhibitory factor [115]. It appears that many of the immunotherapies attempted against advanced epithelial cancers have the capacity to induce the production of potent CTLs, yet this has not proven sufficient to translate to improved survival in all cases, likely as a result of tolerogenic factors within the tumor microenvironment. Recent reports of induction of antitumor immune responses upon combination of CTLA-4 blockade along with vaccination in ovarian cancer patients [116, 117] highlight the relevance of overcoming immunosuppression, particularly in conjunction with other immune strategies to produce antitumor immunity. Therefore, it has become abundantly clear from the wealth of experimental data in this field that due to the diversity of mechanisms employed by tumors to evade immune destruction, the appropriate immunotherapeutic regime may not simply target an individual aspect, but may need to incorporate strategies that address multiple immune pathways.

Several reports propose various methods for enhancing the *in vivo* survival of adoptively transferred lymphocytes. One such method is through the sublethal irradiation of tumor-bearing hosts to create space to accommodate the expansion of the transferred T cells. We found that even under the context of irradiation and depleting regulatory myeloid cells from tumor locations, our transferred T cells did not persist for long periods, although the combination of irradiation and immunosuppressive myeloid cell depletion enhanced the therapeutic benefit observed when T cells were transferred into tumor-bearing mice. As an individual intervention, elimination of immunosuppressive myeloid cells in tumor-bearing mice disrupted tumor vasculature, produced an immunogenic boost, and thereby delayed tumor progression [15]. Accordingly, the elimination of this immunosuppressive population of cells bolstered the *in vivo* expansion and therapeutic effectiveness of adoptive immunotherapy in our ovarian cancer models, but not the persistence of transferred lymphocytes [24, 86]. 

While irradiation did not enhance the survival of the transferred T cells, it likely enhanced the immunogenicity through inducing the death of some tumor cells, and thus releasing tumor antigen that could trigger host immune responses. Furthermore, irradiation can cause upregulation of certain molecules on tumor cells, such as MHC-I or the death receptor Fas, that render them more immunogenic and flag them as better targets for immune elimination [118]. 

The persistence of transferred T cells correlates with greater efficacy in most cancer systems, thus enhancing the survival of these transferred T cells is a future direction to be taken into consideration. Stimulation of CD40 and Toll-like Receptor 3 on ovarian cancer infiltrating DCs converts them from immunosuppressive to immunostimulatory cells and boosts T cell-mediated antitumor immune responses [22]. Such pretreatment of tumor bearing hosts before ACT may extend the survival of transferred T cells. Ongoing studies in our laboratory should define the potential of this approach. 

Notwithstanding, the impact of standard treatment modalities should not be disregarded and immune therapies should probably be administered in conjunction with, rather than, in place of such. Surgical debulking may still be a necessary procedure for the removal of large tumor masses, while, as we and others have demonstrated, chemotherapy/radiation therapy may bolster the effects of immunotherapies. Finally, immune-based therapies may add to the antitumor armament by eradicating residual disease and activating endogenous antitumor responses that persist ideally in the memory compartment to prevent metastatic lesions and to control recurrences.

Such trimodal approaches (surgery plus chemotherapy/radiation plus immunotherapy) probably represent the future in the battle against epithelial cancers. Immunotherapeutic interventions, since largely hypothetical, are tested in patients with late stage, very advanced disease, or recurrent disease that is often refractory to standard therapies, in which case the efficacy of any intervention is highly unlikely and mostly improbable. Trials in patients whose disease has not progressed as far may prove to reveal more favorable clinical outcomes, and, through the elicitation of protective endogenous immune responses, may prevent recurrence and increase the rate of survival of endothelial cancer patients. Drastic measures need to be taken to defeat the grim effects of the most devastating cancers.

## 10. Effect of ACT on Endogenous Ongoing Antitumor Immunity

 The prevailing concept surrounding ACT is that successful ACT requires the persistence of the transferred T cells, which are considered the ultimate mediators of the antitumor response. Importantly, the contribution of endogenous responses to the efficacy of immune-based therapies has been a largely neglected area. As stated above, however, our studies in ovarian cancer models show that such endogenous responses are not only important, but crucial to the elimination of established tumors and the induction of persistent memory responses [24]. As described above, we found that our T cells briefly primed against tumor antigens do not persist for very long (as in human ovarian cancer) but instead elicit the awakening of host immune populations that induce sustained antitumor responses [24, 86]. Existing (although obviously suboptimal) antitumor responses were significantly boosted in mice receiving adoptively transferred tumor-reactive T cells. Most importantly, endogenous T cell-mediated responses were long-lived and more persistent than the activity of transferred lymphocytes. Thus, adoptively transferred T cells stimulate the awakening of host immune responses and host cells after ACT developed the ability to recognize and react to tumor antigens. The transferred T cells required perforin for maximal effectiveness suggesting that these transferred CTLs induce immunogenic tumor death triggering the release of tumor antigen that may prime DC activation. CD4^+^ T cells provided further costimulatory molecules to complete the activation of these DCs indicating that the adoptively transferred CD4^+^ and CD8^+^ T cells cooperate to induce their antitumor effects.

These results imply that while persistence and direct antitumor activity of adoptively transferred T cells is crucial for their therapeutic potential, and how they impact existing immune responses may be another variable to optimize in a clinical context. Unleashing endogenous antitumor immunity may also result from host-conditioning strategies and synergize with ACT. Thus, interventions aimed to transform tumor microenvironmental cells from an immunosuppressive to an immunostimulatory phenotype (such as CD40+TLR agonists) may be ideal to boost the expansion, persistence, and therapeutic activity of both adoptively transferred and endogenous tumor-reactive lymphocytes.

## 11. Concluding Remarks

 Despite a great deal of effort being dedicated to the development of new therapies, there has been minimal improvement in the survival rate for most cancers including epithelial ovarian cancer. Strategies that have proven successful in certain malignancies have not produced similar results in epithelial cancers like ovarian cancer, highlighting the complexities existing within the microenvironment of individual cancers and emphasizing the need to consider each tumor as an independent entity. T cell therapies often fail due to the tolerogenic environment in which the T cells are placed and that integrating techniques that reduce the immunosuppressive nature of the tumor microenvironment will enhance the efficacy of ACT and make it a viable treatment modality. Newly developed immunotherapies will need to address multiple immune pathways and circumvent various mechanisms of immune evasion and importantly need to incorporate strategies that contribute to the induction of endogenous responses which we had found to be not only beneficial, but crucial to the elimination of established tumors and the induction of persistent memory responses. It is apparent that the appropriate T cell polarization and differentiation will need to be identified in individual tumor systems for the optimal function of anticancer lymphocytes, and to break the tumor-induced paralysis of host immune responses. Furthermore, while most studies have focused on the contribution of or administration of cytotoxic CD8^+^ T cells, it is becoming increasingly clear that the coadministration of appropriate CD4^+^ T cell subsets may be advantageous to the therapeutic effects of ACT, particularly through the elicitation of endogenous antitumor responses, and their incorporation into ACT regimens should be further investigated.
